# Oxidative Modification of Blood Serum Proteins in Multiple Sclerosis after Interferon Beta and Melatonin Treatment

**DOI:** 10.1155/2017/7905148

**Published:** 2017-10-18

**Authors:** Monika Adamczyk-Sowa, Sabina Galiniak, Ewa Żyracka, Michalina Grzesik, Katarzyna Naparło, Paweł Sowa, Grzegorz Bartosz, Izabela Sadowska-Bartosz

**Affiliations:** ^1^Department of Neurology in Zabrze, Medical University of Silesia, 3-go Maja St. 13-15, 41-800 Zabrze, Poland; ^2^Department of Histology and Embryology, Chair of the Morphological Sciences, University of Rzeszów, Leszka Czarnego 4, 35-615 Rzeszów, Poland; ^3^Department of Analytical Biochemistry, Faculty of Biology and Agriculture, University of Rzeszów, ul. Zelwerowicza 4, 35-601 Rzeszów, Poland; ^4^Department of Laryngology in Zabrze, Medical University of Silesia, ul. Curie-Skłodowskiej 10, 41-800 Zabrze, Poland; ^5^Department of Molecular Biophysics, Faculty of Biology and Environmental Protection, University of Łódź, Pomorska 141/143, 90-236 Łódź, Poland

## Abstract

Multiple sclerosis (MS) is a disease involving oxidative stress (OS). This study was aimed at examination of the effect of melatonin supplementation on OS parameters, especially oxidative protein modifications of blood serum proteins, in MS patients. The study included 11 control subjects, 14 de novo diagnosed MS patients with the relapsing-remitting form of MS (RRMS), 36 patients with RRMS receiving interferon beta-1b (250 *μ*g every other day), and 25 RRMS patients receiving interferon beta-1b plus melatonin (5 mg daily). The levels of N′-formylkynurenine, kynurenine, dityrosine, carbonyl groups, advanced glycation products (AGEs), advanced oxidation protein products (AOPP), and malondialdehyde were elevated in nontreated RRSM patients. N′-Formylkynurenine, kynurenine, AGEs, and carbonyl contents were decreased only in the group treated with interferon beta plus melatonin, while dityrosine and AOPP contents were decreased both in the group of patients treated with interferon beta and in the group treated with interferon beta-1b plus melatonin. These results demonstrate that melatonin ameliorates OS in MS patients supporting the view that combined administration of interferon beta-1b and melatonin can be more effective in reducing OS in MS patients than interferon beta-1b alone.

## 1. Introduction

Multiple sclerosis (MS) is one of the most widespread chronic inflammatory, demyelinating diseases of the central nervous system (CNS), which leads to damage of myelin and axons. Although the exact cause of MS is unknown, it is considered that genetic predisposition, environmental factors, and abnormal immune response, consisting of delivery of cytokines from lymphocytes including Th1 and Th17 cells, contribute to the pathogenesis of this disease [1, 2]. In recent years, the factors involved in the etiology of the disease have also included oxidative stress (OS), which is defined as an imbalance between the generation of reactive oxygen species (ROS) and the mechanisms that are responsible for their elimination. It is suggested that the increased generation of ROS and reactive forms of nitrogen (RNS) leads to oxidative and nitrosative stress causing damage to mitochondria and myelin, oligodendrocyte apoptosis, and astrocyte dysfunction [3]. Elevated OS markers were observed in the blood, plasma, and cerebrospinal fluid of patients with relapsing-remitting (RRMS) form of MS [4–7] and in the plasma of patients with secondary progressive (SPMS) form of MS [7], which confirms that OS plays a significant role in MS and implies that oxidative damage to blood serum proteins correlates with the severity of disease.

Melatonin (N-acetyl-5-methoxytryptamine; MEL) is a natural hormone derivative of tryptophan. In animals, it is synthesized primarily by pinealocytes of the pineal gland and regulates sleep and wakefulness. Melatonin is known as a scavenger of reactive oxygen and nitrogen species and an agent decreasing ROS generation as well as increasing the activity of antioxidant enzymes and glutathione content [8]. More and more studies have reported that MEL can improve memory impairment as a result of its antioxidant properties, which indicates that MEL may have a beneficial role in the treatment of neurodegenerative disorders involving enhanced OS [9–12].

Furthermore, MEL reduced clinical scores as well as ROS generation and delayed manifestation of motor symptoms in experimental autoimmune encephalomyelitis, which is an often used animal MS model. MEL also restored increased levels of microglia and CD4+ T cells in untreated animals to the control level and decreased the loss of oligodendrocytes, demyelination, and axonal injury [13].

In view of the occurrence and postulated role of OS in MS, examination of the effects of antioxidants in combination with standard treatment seems noteworthy. This study was aimed at examining the effect of MEL on the OS markers in MS patients treated with interferon beta-1b, basing mainly on the oxidative modifications of serum proteins as sensitive markers of OS [14, 15].

## 2. Patients and Methods

### 2.1. Patients

The study was approved by the local Ethics Committee of the Medical University of Silesia (KNW/0022/KB1/130/12). Informed consent was obtained from all individual participants included in the study. After obtaining informed consent, demographic data, Kurtzke's Expanded Disability Status Scale (EDSS) [16], and MRI examinations were performed in all MS patients at the beginning of the study, in accordance with standard clinical protocols. Neurological examination was performed by a qualified neurologist using the EDSS before the therapy and after its completion.

We excluded patients with the following chronic disorders: diabetes, obesity (BMI over 30), hormonal, urinary or liver abnormalities, infectious or inflammatory diseases, dyslipidemia, and smoking. We also excluded patients taking antioxidant substances, vitamins, or anti-inflammatory medications, as well as those who received hormonal treatment within the last 3 months before the study and those who took sleeping medication in the last 2 weeks before the study.

The patients were divided into the following groups:

- Control group consisted of 11 healthy controls observed in the Department of Neurology in Zabrze, Medical University of Silesia, Poland, due to undiagnosed headaches. Controls were matched for age and sex with the study group.
- RRMS untreated group was composed of 14 de novo diagnosed patients, with the relapsing-remitting form of MS (RRMS), according to the McDonald criteria (2005) [17], with immunomodifying pretreatment, but without any immunomodifying MS treatment.
- RRMS INF-beta group was composed of 36 patients with RRMS, diagnosed according to the McDonald criteria. All of them received interferon beta-1b [Betaferon (250 *μ*g injected subcutaneously every other day)].
- RRMS INF-beta + MEL group consisted of 25 RRMS patients receiving interferon beta-1b injected subcutaneously every other day supplemented orally with MEL, 5 mg per day, over a period of 90 days.

Demographic characteristics of the studied groups are presented in Table 1.

### 2.2. Materials

All basic reagents were from Sigma-Aldrich (Poznań, Poland), unless indicated otherwise. Fluorimetric and absorptiometric measurements were done in a Tecan Infinite 200 PRO multimode reader (Tecan Group Ltd., Männedorf, Switzerland) or in an EnVision Multilabel Plate Reader (Perkin-Elmer, Überlingen, Germany). All measurements were performed in triplicate and repeated a minimum of three times.

### 2.3. MRI Examination

Head magnetic resonance imaging (MRI) was performed in all MS patients at the beginning of the study. The 1.5T scanner imaging (General Electric HDx, USA) and standard head protocol for MS patients (multiple planes, slice thickness 5 mm, contrast media: Gadovist [Gd]) and additional postcontrast 3DT1 sequences (1 mm slice thickness) were used. Supratentorial, infratentorial, and number of enhancing T1 plaques were evaluated.

### 2.4. Blood Sampling

Samples of venous blood (10 ml) from MS patients and controls were collected into serum-separating tubes and immediately centrifuged to isolate serum. Collected serum samples were stored at −80°C until biochemical analysis, for not more than 2 months. They were thawed at room temperature only once at the time of analysis.

### 2.5. Estimation of Protein Carbonyls

The content of protein carbonyls was estimated using OxiSelect™ Protein Carbonyl Fluorometric Assay Kit (Cell Biolabs Inc.) according to the protocol supplied by the manufacturer.

### 2.6. Estimation of Protein Oxidative Modifications

Products of oxidative modifications of proteins were estimated on the basis of their characteristic fluorescence. Fluorescence measurements were done by applying 150 *μ*l of the serum diluted 1 : 50 with phosphate-buffered saline (PBS; 1 tablet of PBS/100 ml H_2_O) to wells of a 96-well plate. Fluorescence was measured at wavelengths of 325/440 nm (AGEs), 330/415 nm (dityrosine), 325/434 nm (N′-formylkynurenine), 365/480 nm (kynurenine), and 295/340 nm (tryptophan) [14, 15].

### 2.7. Estimation of Thiol Groups

Thiol groups were estimated using a modification of the Ellman's method [18]. Samples (20 *μ*l) were pipetted to wells of a 96-well plate containing 100 *μ*l of 0.1 M phosphate buffer, pH 8.0. Afterwards, 2 *μ*l of 10 mg/ml Ellman's reagent [5,5′-dithiobis-(2-nitrobenzoic acid); DTNB] was added. Absorbance was measured after 1 h incubation in the dark at 37°C at the wavelength of 412 nm against a reagent blank. The thiol group content was calculated on the basis of a standard curve using glutathione as a standard.

### 2.8. Estimation of Protein

The protein concentration was estimated using the method of Lowry et al. [19]. Serum diluted 200 times with PBS (100 *μ*l) was mixed with 500 *μ*l of the Lowry reagent (formed by mixing 30 ml of 2% Na_2_CO_3_ in 0.1 M NaOH, 0.6 ml of 5% C_4_H_4_O_6_KNa·4H_2_O, and 0.6 ml of 2% Cu_2_SO_4_) and incubated at room temperature for 10 min. Afterwards, 50 *μ*l of the Folin–Ciocalteu reagent was added; the plate was shaken and incubated at room temperature for 30 min. The absorbance was measured at 750 nm. Standard curve was prepared with human serum albumin (0–300 *μ*g/ml).

### 2.9. Estimation of Malondialdehyde (MDA)

The serum samples (50 *μ*l serum plus 50 *μ*l PBS or 100 *μ*l PBS blank) were mixed with ice-cold 200 *μ*l of mixture (1 : 1) of 0.37% thiobarbituric acid (TBA) and 15% trichloroacetic acid (TCA) in 0.25 M HCl to precipitate protein. The reaction was performed at pH 2-3 at 100°C for 40 min. The precipitate was pelleted by centrifugation at 3000 ×g at 4°C for 10 min. Absorbance of supernatants was read at a wavelength of 532 nm.

The majority of TBA-reactive substances (TBARS) is malondialdehyde; thus, the concentration of MDA in blood serum was expressed as *μ*M MDA. The results were calculated using an absorption coefficient for MDA of 1.56 × 10^5^ M^−1^ cm^−1^.

### 2.10. Estimation of AOPP

Advanced oxidation protein products (AOPP) were estimated using the method of Witko-Sarsat et al. [20]. 200 *μ*l of serum diluted 1 : 5 with PBS was applied to a 96-well plate, and 20 *μ*l of acetic acid was added to each well. Absorbance was measured at 340 nm against a blank containing 200 *μ*l of PBS, 20 *μ*l of acetic acid, and 10 *μ*l of 1.16 M potassium iodide. Calibration curve was prepared using chloramine-T at concentrations of 0–100 *μ*M by applying 200 *μ*l chloramine-T, 20 *μ*l acetic acid, and 10 *μ*l of 1.16 M potassium iodide to the plate. AOPP concentration is expressed in nmol chloramine-T-equivalents/mg protein.

### 2.11. Estimation of Total Antioxidant Capacity of Blood Serum as FRAP

Total antioxidant status was measured in serum using the ferric reducing antioxidant power assay (FRAP). The ferric reducing antioxidant potential assay measures the ability of antioxidants to reduce ferric (Fe^3+^) ions to ferrous (Fe^2+^) ions [21]. 0.3 M acetate buffer (pH = 3.6), 0.01 M TPTZ (2,4,6-tripyridyl-s-triazine) in 0.04 M HCl, and 0.02 M FeCl_3_^∗^ 6H_2_O mixed in 10 : 1 : 1 and 180 *μ*l of this mixture were added to wells of a 96-well plate containing 10 *μ*l of sample and 10 *μ*l of PBS. The reduction of Fe^3+^-2,4,6-tripyridyl-s-triazine complex to the ferrous form at low pH was monitored by measuring the absorption change after 20 min incubation at room temperature at 593 nm. The value was calculated relevant to the activity of Trolox and expressed as *μ*moles Trolox equivalents/l (*μ*M).

### 2.12. Estimation of Total Antioxidant Capacity with ABTS^∗^

Antiradical activity is a measure of the ability of a given compound to react with free radicals. One stable free radical employed in such reactions is the 2,2′-azinobis(3-ethylbenzthiazoline-6-sulfonic acid) radical (ABTS^∗^). Standard antioxidants react rapidly with ABTS^∗^ (within seconds; “fast antioxidants”) while some react at a lower rate (“slow antioxidants”) [22]. Briefly, 2 *μ*l of sample and 18 *μ*l of PBS were added to a solution of ABTS^∗^, diluted such that 200 *μ*l of the solution had absorbance of 1.0 in a microplate well. The decrease in ABTS^∗^ absorbance was measured after 1 min (“fast” scavenging) and between 10 and 30 min (“slow” scavenging) of incubation at ambient temperature (21 ± 1°C) at 414 nm. ABTS^∗^ scavenging activity was calculated relevant to the activity of Trolox and expressed as *μ*moles Trolox equivalents/l (*μ*M).

### 2.13. Statistical Analysis

All experiments were performed in triplicate. Data are shown in the form of arithmetic mean values and standard deviations. Statistical analysis was done using one-way analysis of variance (ANOVA/Dunnett's test) for multiple samples and Student's *t*-test for comparing paired sample sets. *p* values less than 0.05 were considered statistically significant. The statistical analysis of the data was performed using STATISTICA (version 12.5, StatSoft Inc. 2XXX, Tulsa, OK, USA, http://www.statsoft.com).

## 3. Results and Discussion

There are several amino acid residues in proteins which are most sensitive to oxidative insult: first of all, cysteine, tryptophan, and tyrosine residues. The decrease in the level of these residues and increase in the level of their modification products are useful biomarkers of OS *in vitro* and *in vivo*.

The level of thiol groups in blood serum reflects predominantly the cysteine thiol groups of serum proteins. The level of thiol groups in the serum, expressed both as thiol concentration and the thiol content of serum proteins, decreased in nontreated patients (from 0.58 ± 0.05 to 0.52 ± 0.09 mM and from 0.52 ± 0.09 to 6.84 ± 0.95 nmol/mg protein, resp.), and the magnitude of this decrease was attenuated in patients treated with INF-beta and INF-beta plus MEL. However, these changes were devoid of statistical significance (not shown).

As a result of OS, the level of tryptophan fluorescence decreases and the levels of products of oxidative destruction of tryptophan such as kynurenine and N′-formylkynurenine, easily detectable by fluorescence, increased.

The values of tryptophan fluorescence decreased in the nontreated patients to 94.9 ± 8.4% of the control value. This decrease was attenuated in patients treated with INF-beta-1b and INF-beta plus MEL, but all these changes lacked statistical significance (not shown).

**Figure 1:**
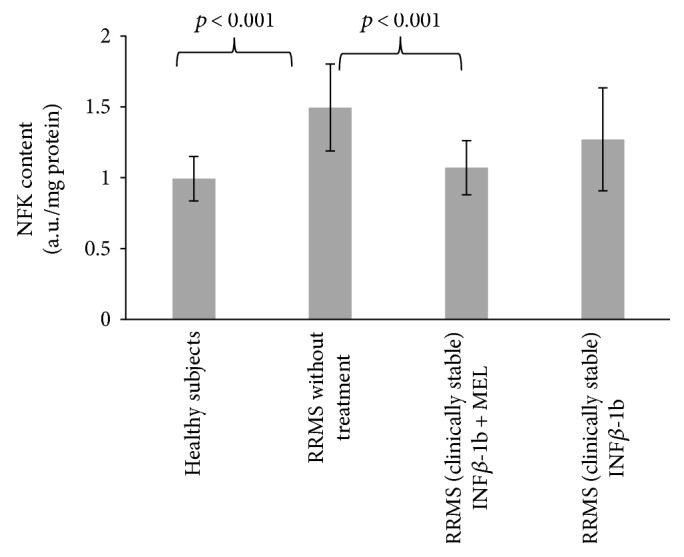
Comparison of N′-formylkynurenine content of blood serum proteins in healthy controls, in RRMS patients without treatment, in RRMS patients treated with INF-beta, and in RRMS patients treated with INF-beta plus MEL. If not indicated, differences are not statistically significant.

The content of N′-formylkynurenine in blood serum proteins of nontreated MS patients was significantly increased with respect to control (*p* < 0.001). Treatment with INF-beta plus MEL prevented this increase (*p* < 0.001), while treatment with INF-beta alone was ineffective (Figure 1).

**Figure 2:**
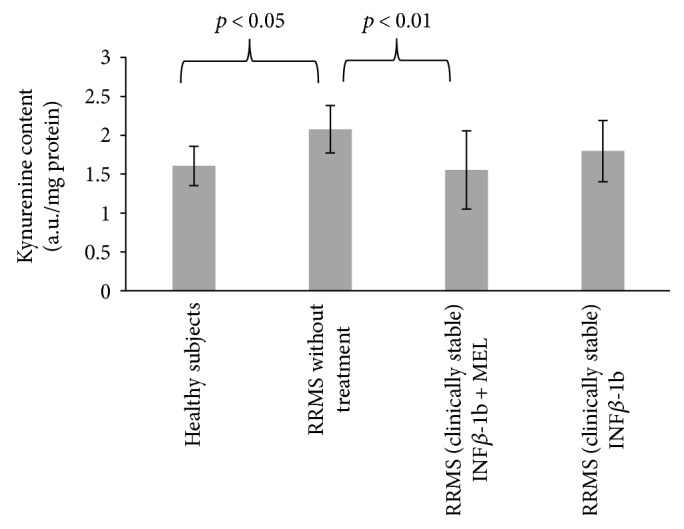
Comparison of kynurenine content of blood serum proteins in healthy controls, in RRMS patients without treatment, in RRMS patients treated with INF-beta, and in RRMS patients treated with INF-beta plus MEL.

The content of kynurenine in blood serum proteins of nontreated MS patients was significantly increased with respect to control (*p* < 0.05). Treatment with INF-beta plus MEL prevented this increase (*p* < 0.01), while treatment with INF-beta alone was again ineffective (Figure 2).

Tyrosine residues are other residues in proteins sensitive to oxidation and also to nitration. Free radical oxidation of tyrosine creates tyrosyl radicals; dimerization of tyrosyl radicals forms dityrosine, which can also be estimated on the basis of its characteristic fluorescence.

**Figure 3:**
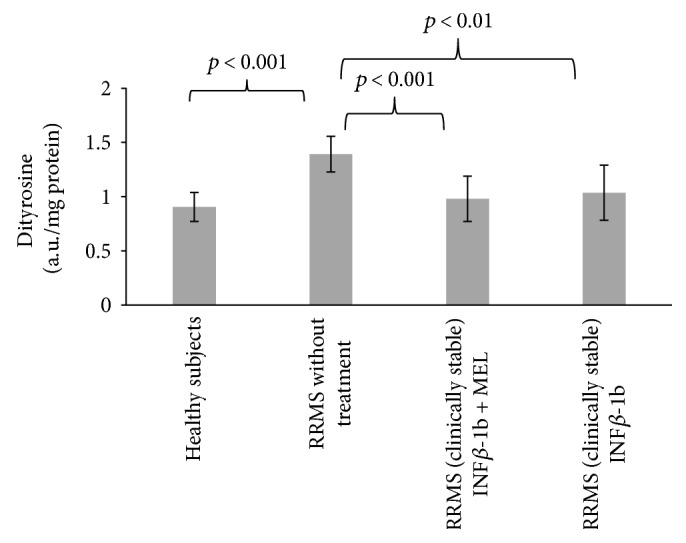
Comparison of dityrosine content of blood serum proteins in healthy controls, in RRMS patients without treatment, in RRMS patients treated with INF-beta, and in RRMS patients treated with INF-beta plus MEL.

The level of dityrosine in blood serum proteins of nontreated MS patients was significantly elevated with respect to control (*p* < 0.001). Treatment with INF-beta (*p* < 0.01) and especially with INF-beta plus MEL (*p* < 0.001) eliminated this elevation (Figure 3).

Protein carbonylation is perhaps the most commonly studied oxidative protein modification induced by ROS. It usually refers to a process that produces reactive ketone or aldehyde residues on proteins that can react with 2,4-dinitrophenylhydrazine (DNPH) to form hydrazones. Protein carbonylation can occur via two ways, as “primary protein carbonylation” by direct oxidation of side chains of some amino acid residues, initiated by ROS and usually metal-catalyzed, and by “secondary protein carbonylation” via addition of aldehydes, generated mainly from lipid peroxidation, such as 4-hydroxynonenal (4-HNE), 2-propenal (acrolein), and malondialdehyde, as well as carbonyl-bearing products of sugar glycoxidation [23, 24].

**Figure 4:**
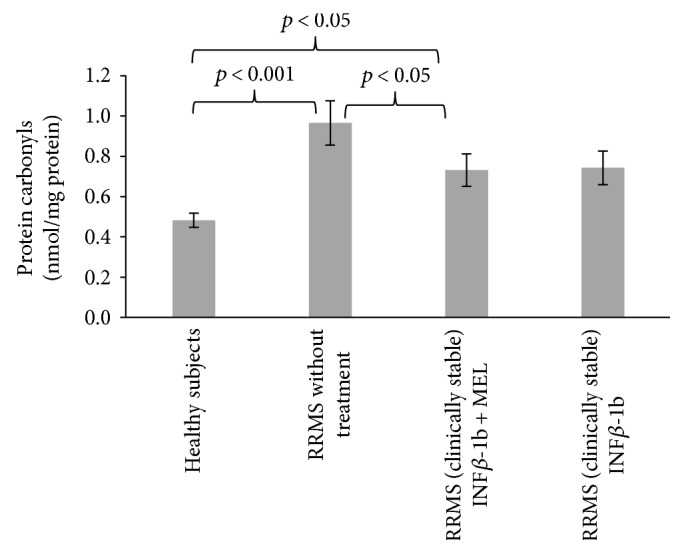
Comparison of carbonyl content of blood serum proteins in healthy controls, in RRMS patients without treatment, in RRMS patients treated with INF-beta, and in RRMS patients treated with INF-beta plus MEL.

The level of protein carbonyls increased in nontreated patients with respect to control (*p* < 0.001); this increase was significantly attenuated (*p* < 0.05) in patients treated with INF-beta plus MEL. The level of protein carbonyls was also decreased in patients treated with only INF-beta, but the decrease was not statically significant with respect to nontreated patients (Figure 4).

**Figure 5:**
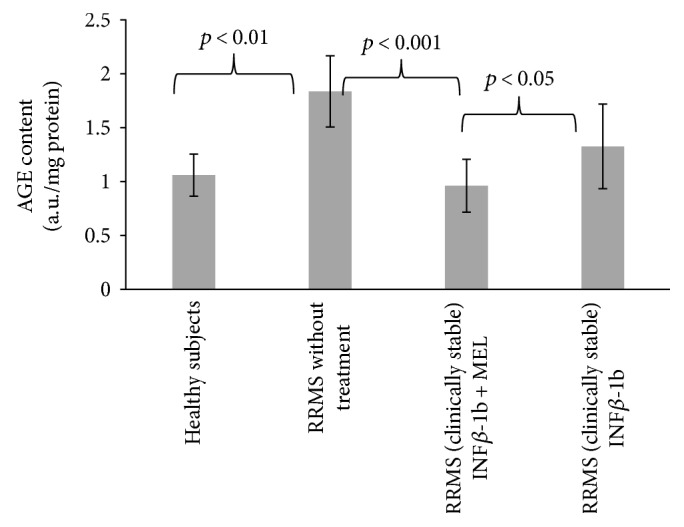
Comparison of AGE content of blood serum proteins in healthy controls, in RRMS patients without treatment, in RRMS patients treated with INF-beta, and in RRMS patients treated with INF-beta and MEL.

Reaction of glycoxidation end products (AGEs) with proteins leads to formation of products with characteristic fluorescence. The fluorescence of AGEs in blood serum proteins of nontreated MS patients was significantly elevated with respect to control (*p* < 0.01). Treatment with INF-beta and MEL prevented this elevation (*p* < 0.001), while the effect of INF-beta alone was devoid of statistical significance (Figure 5).

The reasons for increase in the AGE fluorescence seem to be unclear as the glucose level is not increased in the patients. It may be perhaps attributed to the acceleration of protein glycation by OS demonstrated to occur *in vitro* [25]. The majority of AGEs *in vivo* are mainly formed in a fast reaction of dicarbonyl compounds, such as methylglyoxal (MGO) and glyoxal, with proteins. Furthermore, the main detoxification system of dicarbonyl compounds, the glyoxalase system, seems to be affected in MS patients, which may contribute to high MGO-derived AGE levels [26]. Advanced glycation end products are increased in inflammatory diseases such as atherosclerosis, obesity, and diabetes and also in neuroinflammatory diseases such as Alzheimer's disease and Parkinson's disease. It was reported that AGEs are increased in the plasma and CNS of MS patients [26, 27]. Wetzels et al. [26] suggested that the accumulation of AGEs in the plasma and central nervous system of MS patients compared to healthy controls may contribute to neuroinflammation and the progression of MS.

**Figure 6:**
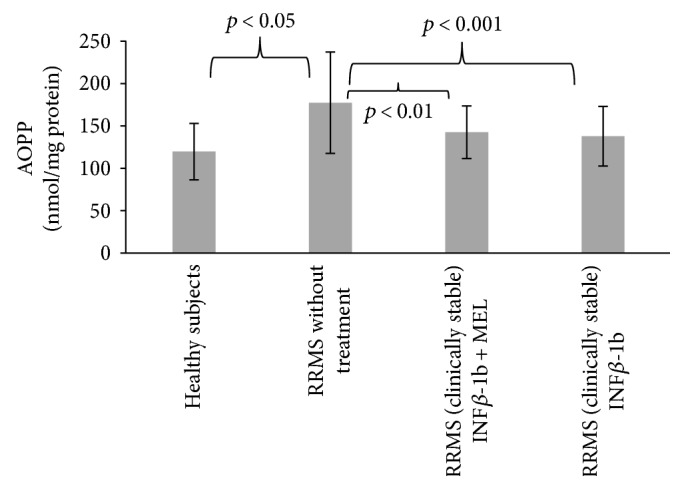
Comparison of AOPP content of blood serum proteins in healthy controls, in RRMS patients without treatment, in RRMS patients treated with INF-beta, and in RRMS patients treated with INF-beta and MEL.

Advanced oxidation protein products (AOPP) consist of oxidized, dityrosine-containing, crosslinked proteins formed mainly by reactions of reactive chlorine species with plasma proteins [28]. The level of AOPP was increased in the nontreated patients (*p* < 0.05); this increase was attenuated in patients treated with INF-beta and INF-beta plus MEL (Figure 6).

Oxidative stress in MS patients was also assessed on the basis of standard parameters such as the level of malondialdehyde (MDA) and total antioxidant capacity of blood serum.

**Figure 7:**
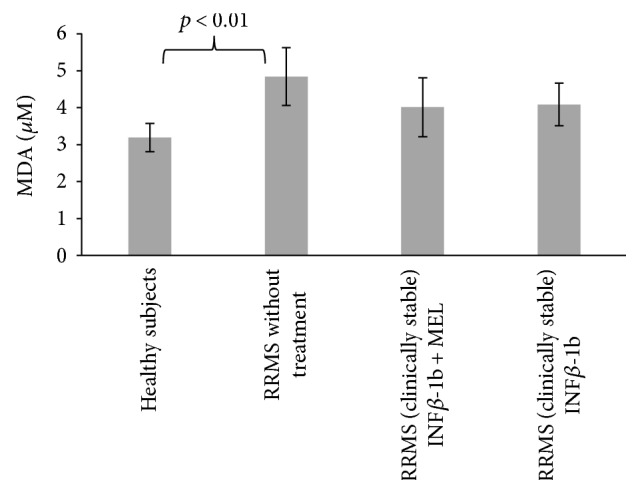
Comparison of malondialdehyde concentration in blood serum proteins in healthy controls, in RRMS patients without treatment, in RRMS patients treated with INF-beta, and in RRMS patients treated with INF-beta and MEL.

The MDA concentration in blood serum was elevated in the nontreated patients, but treatment with either INF-beta or INF-beta plus melatonin did not attenuate this increase (Figure 7).

Blood serum FRAP was decreased in nontreated MS patients from 368.6 ± 77.7 to 318.0 ± 77.8 *μ*M Trolox equivalents; this decrease was attenuated especially in patients treated with INF-beta plus MEL (378.0 ± 112.8 *μ*M Trolox equivalents), but all these changes lacked statistical significance.

**Figure 8:**
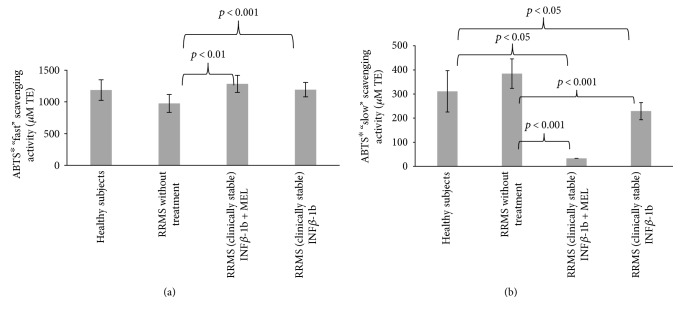
Comparison of “fast” (a) and “slow” (b) ABTS^∗^ scavenging activity of blood serum proteins in healthy controls, in RRMS patients without treatment, in RRMS patients treated with INF-beta, and in RRMS patients treated with INF-beta and MEL.

“Fast” ABTS^∗^ scavenging activity of blood serum was decreased in the patients with respect to control subjects, but this decrease was devoid of statistical activity. However, treatment with INF-beta and with INF-beta plus MEL caused significant elevation of this activity (*p* < 0.001 and *p* > 0.01, resp.). MEL alone could not contribute significantly to the ABTS^∗^ scavenging activity of blood serum as its submicromolar concentrations attainable *in vivo* are insignificant with respect to other contributing compounds [29]. “Slow” ABTS^∗^ scavenging activity was increased in the nontreated patients (devoid of statistical significance); treatment with either INF-beta or INF-beta plus MEL decreased “slow” ABTS-scavenging activity (*p* < 0.001 in both cases; Figure 8).

It is not obvious what determines the “slow” ABTS^∗^ scavenging activity of blood serum. Amino acids such as tryptophan and tyrosine show this type of reactivity [22]. It is possible that the increase in the “slow” ABTS^∗^ scavenging activity may be due to leakage of some intracellular slowly reacting antioxidants.

In summary, the study corroborates the usefulness of protein oxidative modifications as sensitive markers of OS in MS. Our results confirm also previous findings on the occurrence of OS treatment of MS and attenuation of OS by INF-beta treatment, judging from the levels of oxidative modifications of blood serum proteins [14, 15]. The antioxidant effect of INF-beta is due to its immunomodulatory and anti-inflammatory action [1]. Combined treatment with INF-beta and melatonin was more effective in attenuating oxidative stress, being effective in diminishing the increase in the levels of N′-formylkynurenine, kynurenine, carbonyl, and AGE content where treatment with INF-beta alone was ineffective. These results confirm our previous finding on the attenuation of OS in MS patients treated with INF-beta and MEL, on the basis of reduction of lipid plasma hydroperoxide level [30].

It does not seem probable that the attenuation of OS by MEL in MS patients is due to the direct antioxidant activity of this compound, as discussed above. However, MEL is also an indirect antioxidant, acting via stimulation of biosynthesis of antioxidants and other protective proteins, which may be the major mechanisms of its action. MEL was shown to increase the level of expression of genes coding for catalase, MnSOD, and sirtuin 1 [31] and is known to regulate the mitochondrial bioenergetic function [32]. Moreover, MEL directly interferes with the differentiation of T cells, inducing the expression of the repressor transcription factor Nfil3, blocking the differentiation of pathogenic Th17 cells and boosting the generation of protective Tr1 cells via Erk1/2 and the transactivation of the IL-10 promoter by ROR-*α* [33]; MEL inhibits also demyelination and increases remyelination [34]. These multiple effects of MEL may, apart from its antioxidant effects, contribute to its positive effects in MS [1, 30, 33, 35].

## 4. Conclusion

This study demonstrates that MEL administration to MS patients undergoing therapy with INF-beta ameliorates oxidative stress, decreasing the extent of the majority of protein oxidative modifications examined. These results support the view that combined administration of INF-beta and MEL can be more effective in reducing oxidative stress in MS patients than INF-beta alone.

## Figures and Tables

**Table 1:** Demographic data of the subjects studied.

Group	Control	RRMS INF-beta + MEL	RRMS INF-beta	RRMS untreated
Subject number (*n*) (total *n* = 86)	11	25	36	14
Age (years) mean ± SD	34.54 ± 9.6	38.16 ± 8.29	39.49 ± 10.16	40.65 ± 10.01
Female/male number (*n*)	6/5	18/7	26/10	7/7
EDSS mean ± SD	NA	1.85 ± 0.75	2.52 ± 1.14	2.68 ± 1.11
Disease duration (years) mean ± SD	NA	4.89 ± 1.41	6.07 ± 3.97	0.88 ± 0.65
Treatment duration (months) mean ± SD	NA	29.51 ± 5.03	28.18 ± 7.13	NA
Number of T2 brain MRI lesions (mean ± SD)	NA	8.70 ± 7.54	9.18 ± 6.56	11.11 ± 10.94
Number of T1 Gd(+) brain MRI lesions (mean ± SD)	NA	0.52 ± 0.17	0.66 ± 0.65	0.86 ± 0.85

NA: nonapplicable. Data are presented as mean ± SD.
